# Public Water Filtration in Massachusetts

**Published:** 1906-05

**Authors:** 


PUBLIC WATER FILTRATION IN MASSACHUSETTS.

Charles Harrington states that since the public water supplies of Massachusetts are in general very efficiently guarded against pollution at their sources, there is no need of purification before distribution, barring a few exceptions. A "mechanical" filter with alum as a coagulant is employed by the town of Athol on account of the abundance of *Anabena* in its water supply. Reading employs the same kind of apparatus on account of the excessive amount of iron in the water. For the reduction of color, and the disagreeable odors and taste due to the overgrowth of *Anabena* and other organisms, the supply for Milford and Hopedale is subjected to filtration through a sand filter-bed. Some years ago the death rate from typhoid fever in Lowell and Lawrence was demonstrated to be due to the infection of the river by the sewage of various cities and towns. A gravity sand filter-bed of two and one half acres was made. The diminution in the number of bacteria and the chemical changes in the water were very marked. The results due to the change in the character of the water supply on the health of the people has been striking. The writer concludes his paper with some interesting statistics relating to this subject.—*Medical Record,* March 24, 1906.

